# Th1/Th2 cytokine pattern in bronchoalveolar lavage fluid and induced sputum in pulmonary sarcoidosis

**DOI:** 10.1186/1471-2466-5-8

**Published:** 2005-06-24

**Authors:** Ioanna Tsiligianni, Katerina M Antoniou, Despina Kyriakou, Nikolaos Tzanakis, George Chrysofakis, Nikolaos M Siafakas, Demosthenes Bouros

**Affiliations:** 1 Department of Pneumonology, University of Crete, Heraklion, Greece; 2 Department of Hematology, University of Thessaly, Larissa, Greece; 3 Department of Pneumonology, Democritus University of Thrace, Alexandroupolis, Greece

## Abstract

**Background:**

Sarcoidosis is thought to be a T-helper type 1 cytokine (Th2 cytokine) mediated disorder. Induced sputum (IS) has been proposed as a useful non-invasive method, mainly for the assessment of the airway diseases. The aim of this study was to explore induced sputum (IS) CD4^+^Th1 T-lymphocyte subpopulation and to compare them with those of bronchoalveolar lavage fluid (BALF) in patients with sarcoidosis.

**Methods:**

We studied prospectively 21 patients (12 female, 9 male) of median age 46 yr (range, 25–65) with sarcoidosis and 10 normal subjects (5 female, 5 male) of median age 39 yr (range, 26–60). IS was performed with hypertonic saline solution using an ultrasonic nebulizer. BALF was performed within 10 days of IS. After stimulation of sputum lymphocytes with phorbol-myristate-acetate, we used double immunocytochemical methods to identify CD4^+ ^IFN-γ positive and IL-4 positive cells (Th1 and Th2, respectively).

**Results:**

Sarcoidosis patients had an increased number of CD4+ -IFN-γ producing cells in IS (p = 0.003) and BALF (p = 0.01) in comparison with normal subjects. No significant differences were detected between CD4+ -IL-4 cells in BALF (p = 0.053, NS) and IS (p = 0.46, NS) between sarcoidosis patients and healthy controls. The ratio of Th1 to Th2 cells in BALF and IS was statistically different in sarcoidosis when compared with normal subjects (p = 0.007 in BALF and IS). A significant correlation was found between CD4+ IFN-γ positive cells in IS and those in BALF in sarcoidosis patients (r = 0.685, p = 0.0006).

**Conclusion:**

These data suggests that a Th1-like cytokine pattern can be observed in CD4+ T-lymphocytes in IS in patients with pulmonary sarcoidosis. Further studies are needed to explore the value of IS vs BALF in the follow-up of these patients.

## Background

Sarcoidosis is a chronic, systemic disease of unknown origin, characterized by the formation of noncaseating granulomas in affected organs, most commonly the lung. According to the ATS/ERS/WASOG [1] statement on sarcoidosis, the diagnosis is based on clinical and radiological findings and on histologic evidence. The technical availability of the fiberoptic bronchoscope has facilitated the development of the bronchoalveolar lavage procedure, which is useful for the collection of cells and secretions from the lower respiratory tract. Although this technique causes low risk to the patient, it is relatively invasive and inconvenient for the patients. Sputum induction has been proposed as a non-invasive method, useful to the investigation of asthma [3], and also of other interstitial lung disease (ILD) [4,5], more specifically sarcoidosis [4,6-9], pneumoconiosis due to dust exposure [10], Crohn's disease [11] and non-granulomatous ILD [4]. The exact role of induced sputum in the diagnosis and evaluation of disease activity in ILD and especially in sarcoidosis has not been clearly defined. Furthermore, no specific studies have been undertaken to evaluate the safety and functional effects of induced sputum in patients with ILD [5]. Today's attitude and opinion of researchers in this area is that induced sputum is a promising technique in assessing ILD, but its diagnostic role has not yet been clarified, and should be used as a complimentary tool to BAL for research and clinical monitoring [5,12].

The pathogenesis of pulmonary sarcoidosis has been related to an increased production of Th1-like cytokines. However cytokine expression in sarcoidosis has not been systematically studied yet. It is well known that CD4^+ ^T cells can be divided in two subgroups on the basis of their cytokines profile. Th1 cells produce type 1 cytokines {IL-2, tumor necrosis factor-(TNF)-α, interferon gamma-(IFN-γ)}, while Th2 cells produce type 2 cytokines (IL-4, IL-5, IL-6, IL-10, IL13).

As far as we know there are only a few studies regarding the Th1/Th2 balance in sarcoidosis and all of them either in bronchoalveolar lavage fluid (BALF) or in blood [13-15]. Therefore, the aim of this study was to explore induced sputum (IS) CD4^+ ^Th1 T-lymphocyte subpopulation and to compare them with those in the BALF in patients with sarcoidosis.

## Methods

### Subjects

The demographic and clinical characteristics of the patients and controls are shown in Table 1. Twenty-one consecutive sarcoidosis patients, 9 male and 12 female, of median (range) age 46 (25–65) years who were investigated in the sarcoidosis clinic of our Hospital were enrolled in the study. Three of them were smokers and 18 non-smokers. The ATS/ERS/WASOG statement [1] on sarcoidosis was followed for the diagnosis, based on history, clinical symptoms, standard chest radiography, computed tomography, lung Ga^67 ^scintigraphy and laboratory tests (serum angiotensin-converting enzyme). All of them had transbronchial or open lung biopsy with histopathological evidence of noncaseating epithelioid cell granulomas.

**Table 1:** Demographic and spirometric characteristics of sarcoidosis patients and control subjects. Values are expressed as mean ± SD, and age as median (range).

**Characteristics**	**Sarcoidosis**	**Normal subjects**
Number	21	10
Sex: Male/Female	12/9	5/5
Age, yr	46 (25–65)	39 (26–60).
Smokers/non smokers	18/3	0/10
FVC, % pred	93.1 ± 6.4	103 ± 13.7
FEV_1_, % pred	92.3 ± 6.6	101.1 ± 19
FEV_1 _/ FVC, % pred	95.3 ± 5.9	100.3 ± 8.6
K CO % pred	82.3 ± 8.1	96 ± 6.3
Radiographic Stage (n)	I: 8, II: 7,III: 6	

Abbreviations: KCO = Carbon monoxide transfer coefficient, FVC % pred : Forced vital capacity % predicted, FEV1 % pred : Forced expiratory volume within the first second % predicted

According to chest radiography classification of sarcoidosis, 8 had type I disease (lymphadenopathy alone, 7 type II disease (lymphadenopathy and parenchymal opacities), and 6 type III disease (only parenchymal opacities).

The control group included 10 healthy nonsmoker volunteers (5 female, 5 male), median (range) age 39 (26 – 60) years who were able to produce adequate sputum samples following sputum induction. Patients and controls with acute respiratory infection during the 6 weeks prior to the study were excluded. The Ethics committee of our hospital approved the protocol and all patients and controls gave their consent.

### Spirometry

Spirometry was performed with a computerized system (MasterLab, Jaeger2.12, Germany). The measurement was performed using standard protocols according to ATS guidelines [16].

### Sputum induction

Sputum was induced by the inhalation of hypertonic saline aerosol solution, generated by an ultrasonic nebulizer (Ultraneb 2000, DeVilbiss, Somerset, PA, USA), as previously described [6,17,18].

### Sputum processing

Sputum was processed within 1 hour after termination of the induction. The method of sputum examination described earlier [17,18] was used with some modifications [6].

### Bronchoalveolar lavage

Fiberoptic bronchoscopy with BAL was performed within two weeks from the IS, as part of routine clinical management, according to recommended guidelines and previous reports [6,20].

### BALF processing

The recovered BAL fluid was filtered through sterile gauze (Thompson, Ontario, Canada) and centrifuged at 400 g for 15 minutes at 4°C. Total cell counts were determined using an improved Neubauer counting chamber, and expressed as the total number of cells per mL of aspirated fluid. The pellet was washed three times with cold PBS-Dulbecco's and the cells were adjusted to a final concentration of 10^6^cells/mL with RPMI1640 plus 2%FCS. The slide preparation was performed as previously reported [6].

### Immunocytochemical analysis

The immunocytochemical analysis and T-cell determination were performed in sputum cytospins. Briefly, sputum lymphocytes were stimulated by incubating suspension B in 24-well plates at a concentration of 2 × 10^3 ^cells/μL for 5 h, under 5% CO_2_, at 37°C, in RPMI-1640 at 10% FCS in the presence of phorbol 12-myristate 13 acetate (PMA) 25 ng/mL, ionomycin 1 μmoL and Brefeldin A 10 μg/Ml (Sigma-AldrichCorp. St.Louis, MO, USA). Cytospins were made using cytocentrifugation of 50 μL of the stimulated suspension and were stored at -80°C for immunocytochemical analysis later. Approximately 175,000 cells were cytospined on each slide (3500 cells/μl × 50 μl = 175,000 cells), among which there was a sufficient number of lymphocytes to stain.

After defrosting the slides, they were fixed in acetone for 10 min and rehydrated in Tris-Buffered Saline (TBS). The double immunocytochemical method was performed in two steps as previously described [21]. Briefly, the specimens were first incubated for 30 min with bovine serum to block the unspecific binding and then were exposed to the first primary antibody at a dilution of 1:50 for 30 min at room temperature. After washing inTBS three times they were exposed to the first secondary antibody, rabbit anti mouse immunoglobulin fluorescein isothiocyanate (FITC)-conjugated for 15 min (ImmunotechMarseille, France) at a dilution of 1:50. After washing in TBS three times they were exposed to the secondary primary antibody at a dilution of 1:50 for 30 min at room temperature. After washing in TBS three times they were exposed to the second secondary antibody, rabbit anti-mouse immunoglobulin phycoerythrin-conjugated (IgG-PE) for 15 min (Immunotech Marseille, France) at a dilution of 1:50. After washing in TBS three times the slides were mounted with 30% glycerol in TBS. Two investigators examined the slides under ultraviolet microscope and their results were averaged. Three replicate measurements were performed by each observer in 10 slides. Both intra- and inter- observer coefficient of variation were <15%. The ratio of CD4^+^-IFN-γ positive cells to CD4^+^-IL4 positive cells was calculated. For the estimation of each ratio, 500 T-cells were counted with more than one cytospin stained if necessary, because this number was sufficient to obtain a mean value per subject that remained constant after further increasing the number of cells counted. Results were also expressed as percentage of lymphocytes by dividing the number of lymphocytes stained per slide by total the number of lymphocytes per slide. Negative controls were obtained by the use of mouse-anti-mouse immunoglobulin to estimate the non specific binding. Positive controls were obtained by the use of T-lymphoma cells cytospined on slides. Macrophages were excluded from counting by morphology.

### Measurement of CD4^+^-IFN-γ and CD4^+^-IL4 producing T-cells

The primary anti CD4 mouse anti-human monoclonal antibody with secondary rabbit anti-mouse IgG- FITC antibody and the primary anti-IFN-γ mouse antihuman monoclonal antibody (Caltag Burlingame, CA, USA) with secondary rabbit antimouse IgG-phycoerythrin-conjugated (IgG-PE) antibody were used. At least 500 CD4^+ ^cells were counted to estimate the number of CD4^+^-IFN-γ cells. Also the same method was applied for staining CD4^+^-IL-4 producing cells with anti-IL4 antibody (Caltag Burlingame, CA, USA).

### Statistics

All analyses were performed using the statistical software StatsDirect for Windows version2.4.1 (Camcode; Cambridge, UK). Results are expressed as mean ± SD, or median (range), unless otherwise indicated. The *Shapiro-Wilk W *test for normality was applied to assess normality. Differences between sarcoidosis patients and controls were tested usingt-test for normally and the Mann-Whitney U test for non normally distributed data. Differences between the BALF and sputumwithingroupswere tested using the paired student's t-test for normally and theWilcoxon's signed rank test for non-normally distributed variables. Correlation between cell numbers and T lymphocyte subsets were analyzed using the Pearson's correlation coefficient. A p value of <0.05 was considered as statistically significant

## Results

Patients with sarcoidosis were older than normal control subjects and had lower FVC, and FEV_1 _values but the differences were not statistically significant. Induced sputum and BALF were tolerated well by all subjects, without any adverse events (Table 1).

In BALF the percentage of IFN-γ producing CD4+ Tcells was significantly higher in patients with sarcoidosis than in normal control subjects (mean ± SD, 53.1 ± 17.9 versus 38.1 ± + 7.7, p = 0.019) after the stimulation with PMA/ionomycin. In induced sputum the percentage of IFN-γ producing CD4+ T cells was significantly higher in patients with sarcoidosis than in normal control subjects (mean ± SD, 60.0 ± 23.3 versus 35.60 ± 12.46, p = 0.003) after the stimulation. No significant differences were detected between sarcoidosis patients and control subjects regarding the proportion of IL-4 producing CD4+ T cells in BALF (p = 0.46) as well as in induced sputum (p = 0.055) (Table 2). The ratio Th1/Th2 was found significantly higher in BALF (p = 0.007) and also in induced sputum compared with healthy controls.

**Table 2:** Mean ± SD of Th1, Th2 lymphocyte subsets in induced sputum (IS) and bronchoalveolar lavage fluid (BALF) in sarcoidosis patients and control subjects.

Induced sputum			BALF	
	Th1	Th2	Th1	Th2

Control	35.6 ± 12.4	0.2 ± 0.15	38.1 ± 7.7	0.3 ± 0.14
Sarcoidosis patients	60.0 ± 23.3	0.2 ± 0.12	53.1 ± 17.9	0.2 ± 0.11
*P*	0.003	0.46	0.019	0.55

* Paired t-test or Wilcoxon's signed rank test, unadjusted p-values.

**Figure 1:**
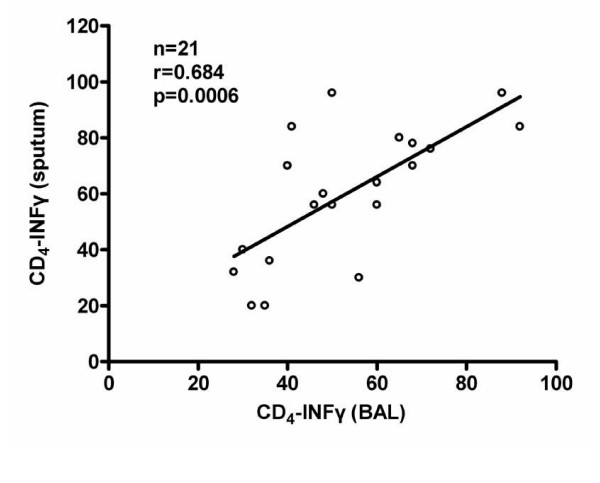
Correlation of CD4+ IFN-γ cells between bronchoalveolar lavage fluid (BALF) and induced sputum (IS).

No statistically significant difference was found between induced sputum and BALF within groups (paired t test). A significant correlation was found between BALF and IS in the percentage of IFN-γ producing CD4+ T cells counts (r= 0. 685, p = 0.006) (Figure 1).

## Discussion

To the best of our knowledge this is the first study in the literature to investigate the balance Th1/Th2 in sarcoidosis with the method of induced sputum. The main finding of this study is that a Th1-like cytokine pattern can be observed in CD4+ BALF as well as induced sputum T lymphocytes. We also detected a positive correlation between CD4+ IFN-γ producing T cells in induced sputum and those in BALF in sarcoidosis patients (p = 0.0006, r = 0.685).

We have previously shown that inflammation in sarcoidosis could be effectively and non-invasively determined by the analysis of cell differential counts and T-lymphocyte subsets in induced sputum [6]. We demonstrated that induced sputum can discriminate between patients with sarcoidosis and normal subsets on the basis of the profile of airway inflammatory cells, including lymphocytes [6]. Based on our findings that there is a positive correlation between BALF and induced sputum lymphocyte counts (r = 0.616) and CD4+/CD8+ ratio (r = 0.7) we aimed to evaluate the Th1/Th2 balance in sarcoidosis in both BALF and induced sputum. As it has already been reported, the mean CD4+/CD8+ ratio was not significantly different between BALF and induced sputum in 20 sarcoidosis patients (p > 0.253) and in normal controls (p > 0.3). In our study population a CD4+/CD8+ ratio ≥ 2.5 was found on 17 patients in induced sputum, while a CD4+/CD8+ ratio > 4 was found in 12 (60%) patients [6].

The balance between Th1 and Th2 helper cells and their associated cytokine patterns can in an immune response influence the phenotype and progression of several clinical diseases. An imbalance in the expression of Th1 and Th2 type cytokines by alveolar cells is thought to play an important role in the immunopathogenesis of sarcoidosis [22-26]. Previous authors have shown a spontaneous release from alveolar cells of the Th1 type cytokines IFN-γ and IL-2 but not Th2 type cytokines [24,26-29]. Additionally, there are no data on cytokine profiles in the late stages of fibrotic sarcoidosis to assess the contribution of Th1 or Th2 cytokines to the fibrotic process. Experimental models confirm that Th2-mediated granulomatous responses are more fibrotic than Th1-mediated inflammation, so in the absence of human data, there is uncertainty as to the relevant immune processes in fibrotic pulmonary sarcoidosis. Conceivably, patients with fibrotic sarcoidosis are those who switch to a more fibrotic Th2 response later in the course of the disease, or have a persistent dominant Th2 response from the iniatial stages of disease [29,30]. Furthermore, data support the immunopathogenetic concept of compartmentalization and the predominance of CD4+ T cells producing mainly Th1 type cytokines in acute pulmonary sarcoidosis, which becomes less prominent during the course of the disease [15]. However, in a study using T cell clones from BALF, Baumer et al. found equal levels of Th1 and Th2 cytokine gene expression in patients with sarcoidosis [31]. Thus, the purpose of the present study was to further investigate the phenotype of T lymphocytes in BALF and in induced sputum in patients with pulmonary sarcoidosis with focus on expression of markers indicating Th1 and Th2 cellular function. Our findings suggest a shift toward a type-1 phenotype in CD4+ T-cell populations in accordance with previous studies in BALF and in peripheral blood [13-15,32,33]. Intracellular cytokine staining could be useful to study cytokine expression in sarcoidosis [6,14]. These data are in agreement with those investigating the cytokine profile of T cell subsets with other methods like flow cytometry [13,32,33]. Inui et al. [13] investigated the alteration of the Th1/Th2 and Tc1/ Tc2 balance in sarcoidosis, using cytokine flow cytometry. The authors showed for the first time a significantly higher percentage of IFN-γ producing CD4+ T cells together with a markedly increased ratio of IFN-γ/IL-4-producing CD4+ T cells in BALF, after PMA/ ionomycin stimulation in patients with sarcoidosis compared with normal subjects. On the other hand, there were no differences in the percentages of IFN-γ or IL-4- producing CD8+ T cells in either the peripheral blood or BALF between patients and controls. In addition, a recent report in eighteen patients with untreated sarcoidoisis, examined the expression of Th1 associated chemokine and cytokine receptors CXCR3, CCR5, and interleukin IL-12R, IL-18R, respectively, as well as of the Th2 associated chemokines receptors CCR4 and CXCR4 on CD4+ and CD8+ T cells [33]. That report showed the lung accumulation of the above Th1 associated receptors, in agreement with recent data of our group [34,35].

## Conclusion

This report is the first to evaluate the critical balance of Th1 to Th2 cells with the method of Induced sputum. In conclusion, these data demonstrate that there are increased numbers of CD4+ IFN-γ producing T cells in induced sputum from patients with pulmonary sarcoidosis, in accordance with the shift towards the Th1 response known to exist in BALF and peripheral blood. The positive correlation between the CD4+ IFN-γ producing T cells in BALF and in induced sputum, suggest that sarcoidosis patients could be followed up with this noninvasive method which has to be investigated in further studies.

## Abbreviations

BALF: Bronchoalveolar Lavage Fluid, FEV1 % pred: Forced expiratory volume within the first second % predicted, FVC % pred: Forced vital capacity % predicted, ILD: Interstitial Lung Disease, KCO= Carbon monoxide transfer coefficient, TNF: Tumor Necrosis Factor, IFN-γ: Interferon gamma, IS: Induced Sputum, TBS: Tris-Buffered Saline.

## Competing interests

This study supported by a grant from the Society for Respiratory Research at the University of Thrace

## Authors' contributions

IT, KMA and DB were involved with the study conception. IT, NT, GC and KMA carried out the data acquisition and interpretation. DK performed the immunocytochemical analysis. NT performed the statistical analysis. KMA and DB prepared the manuscript. DB and NMS were involved in revising the article for important intellectual content. All authors read and approved the final manuscript.

## Pre-publication history

The pre-publication history for this paper can be accessed here:



## References

[B1] Hunninghake GW, Costabel U, Ando M, Baughman R, Cordier JF, du Bois R, Eklund A, Kitaichi M, Lynch J, Rizzato G, Rose C, Selroos O, Semenzato G, Sharma OP (1999). ATS/ERS/WASOG statement on sarcoidosis. American Thoracic Society/European Respiratory Society/World Association of Sarcoidosis and Granulomatous Disorders. Sarcoidosis Vasc Diffuse Lung Dis.

[B2] Costabel U, Guzman J (2001). Bronchoalveolar lavage in interstitial lung disease. Curr Opin Pulm Med.

[B3] Fahy JV, Wong H, Liu J, Boushey HA (1995). Comparison of samples collected by sputum induction and bronchoscopy from asthmatic and healthy subjects. Am J Respir Crit Care Med.

[B4] Fireman E, Topilsky I, Greif J, Lerman y, Schwarz Y, Man A, Topilsky M (1999). Induced sputum compared to bronchoalveolar lavage for evaluating patients with sarcoidosis and non-granulomatous interstitial lung disease. Respir Med.

[B5] Olivieri D, D'Ippolito R, Chetta A (2000). Induced sputum: diagnostic value in interstitial lung disease. Curr Opin Pulm Med.

[B6] Tsiligianni I, Tzanakis N, Kyriakou D, Chrysofakis G, Siafakas N, Bouros D (2002). Comparison of sputum induction with bronchoalveolar lavage cell differential counts in patients with sarcoidosis. Sarcoidosis Vasc Diffuse Lung Dis.

[B7] D'Ippolito R, Chetta A, Olivieri D (2000). Role of induced sputum in interstitial lung disease. Eur Respir J.

[B8] D'Ippolito R, Foresi A, Chetta A, Casalini A, Castagnaro A, Leone C, Olivieri D (1999). Induced sputum in patients with newly diagnosed sarcoidosis: comparison with bronchial wash and BAL. Chest.

[B9] Vassilakis DA, Sourvinos G, Pantelidis P, Spandidos DA, Siafakas NM, Bouros D (2001). Extended genetic alterations in a patient with pulmonary sarcoidosis, a benign disease. Sarcoidosis Vasc Diffuse Lung Dis.

[B10] Fireman E, Greif J, Schwarz Y, Man A, Ganor E, Ribak Y, Lernan Y (1999). Assessment of hazardous dust exposure by BAL and induced sputum. Chest.

[B11] Fireman Z, Osipov A, Kivity S, Kopelman Y, Sternberg A, Lazarov E, Fireman E (2000). The use of induced sputum in the assessment of pulmonary involvement in Crohn's disease. Am J Gastrenterol.

[B12] Fireman E (2001). Induced sputum: opening a new window to the lung. Sarcoidosis Vasc Diffuse Lung Dis.

[B13] Inui N, Chida K, Suda T, Nakamura H (2001). Th1/Th2 and Tc1/Tc2 profiles in peripheral blood and bronchoalveolar lavage fluid cells in pulmonary sarcoidosis. J Allergy Clin Immunol.

[B14] Prasse A, Georges CG, Biller H, Hamm H, Mathys H, Luttman W, Virchow JC (2001). Th1 cytokine pattern in sarcoidosis is expressed by bronchoalveolar CD4 and CD8 T cells. Clin Exp Immunol.

[B15] Mollers M, Aries SP, Dromann D, Mascher B, Braun J, Dalhoff K (2001). Intracellular cytokine repertoire in differentT cell subsets from patients with sarcoidosis. Thorax.

[B16] American Thoracic Society (1995). Standardization of spirometry, 1994 update. Am J Respir Crit Care Med.

[B17] Popov T, Pizzichini M, Pizzichini E, Kolendowicz R, Punthakee Z, Dolovich J, Hargreave F (1995). Some technical factors influencing the induction of sputum for cell analysis. Eur Respir J.

[B18] Popov T, Gottschalk R, Kolendowicz R, Dolovich J, Powers P, Hargreave F (1994). The evaluation of a cell dispersion method of sputum examination. Clin Exp Allergy.

[B19] Kips JC, Fahy JV, Hargraeve FE, Ind PW, Veen JC (1998). Methods for sputum induction and analysis of induced sputum: a method for assessing airway inflammation in asthma. Eur Respir J.

[B20] Report of the European Society of Pneumology Task Group (1989). Technical recommendations and guidelines for bronchoalveolar lavage (BAL). Eur Respir J.

[B21] Kyriakou D, Alexandrakis MG, Kyriakou ES, Liapi D, Kourelis TV, Mavromanolakis M, Vlachonikolis I, Eliakis P (2000). Aberrant expression of the major sialoglycoprotein (CD43) on the monocytes of patients with myelodysplastic syndromes. Ann Hematol.

[B22] Muller-Quernheim J (1998). Sarcoidosis: immunopathogenetic concepts and their clinical application. Eur Respir J.

[B23] Agostini C, Costabel U, Semenzato G (1998). Sarcoidosis news: immunologic frontiers for new immunosuppressive strategies. Clin Immunol Immunopathol.

[B24] Walker C, Bauer W, Braun RK, Menz G, Braun P, Schwarz F, Hansel TT, Villiger B (1994). Activated T cells and cytokines in bronchoalveolar lavages from patients with various lung diseases associated with eosinophilia. Am J Respir Crit Care Med.

[B25] Drent M, Grutters JC, Mulder PG, van Velzen-Blad H, Wouters EF, van den Bosch JM (1997). Is the different T helper cell activity in sarcoidosis and extrinsic allergic alveolitis also reflected by the cellular Bronchoalveolar lavage fluid profile?. Sarcoidosis Vasc Diffuse Lung Dis.

[B26] Minshall EM, Tsicopoulos A, Yasruel Z, Wallaert B, Akoum H, Vorng H, Tonnel AB, Hamid Q (1997). Cytokine mRNA gene expression in active and nonactive sarcoidosis. Eur Respir J.

[B27] Hoshino T, Itoh K, Gouhara R, Yamada A, Tanaka Y, Ichikawa Y, Azuma M, Mochizuki M, Oizumi K (1995). Spontaneous production of various cytokines except IL-4 from CD4+ T cells in the affected organs of sarcoidosis patients. Clin Exp Immunol.

[B28] Moller DR, Forman JD, Liu MC, Noble PW, Greenlee BM, Vyas P, Holden DA, Forrester JM, Lazarus A, Wysocka M, Trinchieri G, Karp C (1996). Enhanced expression of IL-12 associated with Th1 cytokine profiles in active pulmonary sarcoidosis. J Immunol.

[B29] Moller DR (1999). Cells and cytokines involved in the pathogenesis of sarcoidosis. Sarcoidosis Vasc Diffuse Lung Dis.

[B30] Moller DR (2003). Pulmonary fibrosis of sarcoidosis. New approaches, old ideas. Am J Respir Cell Mol Biol.

[B31] Baumer I, Zissel G, Schlaak M, Muller-Quernheim J (1997). Th1/Th2 cell distribution in pulmonary sarcoidosis. Am J Respir Cell Mol Biol.

[B32] Wahlstrom J, Katchar K, Wigzell H, Olerup O, Eklund, Grunewald J (2001). Analysis of intracellular cytokines in CD4+ and CD8+ lung and blood T cells in sarcoidosis. Am J Respir Crit Care Med.

[B33] Katchar K, Eklund A, Grunewald J (2003). Expression of Th1 markers by lung accumulated T cells in pulmonary sarcoidosis. Journal of InternalMedicine.

[B34] Antoniou KM, Alexandrakis M, Sfiridaki K, Tzanakis N, Symvoulakis EK, Tsiligianni I, Bouros D, Siafakas NM (2004). Comparison of sputum induction with Bronchoalveolar Lavage Fluid Cytokine IL-12 and IL-18 levels in patients with idiopathic pulmonary fibrosis (IPF/UIP). Am J Respir Crit Med.

[B35] Antoniou KM, Tsiligianni I, Alexandrakis, Sfiridaki K, Tzortzaki EG, Tzanakis N, Bouros D, Siafakas NM (2004). Th1 cytokine profile pattern in peripheral blood, induced sputum and Bronchoalveolar lavage fluid in pulmonary sarcoidosis. Am J Respir Crit Med.

